# Menopausal Hormone Therapy, an Ever-Present Topic: A Pilot Survey about Women’s Experience and Medical Doctors’ Approach

**DOI:** 10.3390/medicina60050774

**Published:** 2024-05-07

**Authors:** Carmen Imma Aquino, Viviana Stampini, Elena Osella, Libera Troìa, Clarissa Rocca, Maurizio Guida, Fabrizio Faggiano, Valentino Remorgida, Daniela Surico

**Affiliations:** 1 Department of Translational Medicine, University of Piemonte Orientale, Gynecology and Obstetrics, “Maggiore della Carità” Hospital, 28100 Novara, Italy; 2 Department of Internal Medicine and Therapeutics, University of Pavia, 27100 Pavia, Italy; 3 Department of Neurosciences and Reproductive Sciences, University of Naples Federico II, 80131 Naples, Italy; 4 Department for Sustainable Development and Ecological Transition, University of Piemonte Orientale, 13100 Vercelli, Italy

**Keywords:** menopause, hormone replacement treatment, endocrine and vasomotor disturbances

## Abstract

*Background and Objective*: Menopause can be associated with many clinical manifestations: vasomotor symptoms, urogenital problems, and additional psychological disturbances, such as anxiety, mood changes, and sleep alterations. The prolonged lack of hormones also increases the risk of long-term consequences. Hormone Replacement Treatment (HRT) in menopause consists of the administration of estrogen, alone or associated to progesterone, to relieve these uncomfortable disturbances and to prevent the onset of other pathologic conditions. The aim of this study is to examine the prevalence of HRT use in a sample of menopausal women and their experience with menopause and HRT. This study also investigates the knowledge of general practitioners (GPs) and gynecologists about HRT and its prescription. *Materials and Methods*: We conducted a cross-sectional population survey on 126 women of 50–59 years in an industrial city in the North of Italy, Vercelli (Novara), in Eastern Piedmont. We also presented a questionnaire on the topic to 54 medical doctors (GPs and gynecologists) of the same area. *Results*: The prevalence of HRT use in our sample was 11.9%. In total, a good percentage of the users affirmed to be satisfied with HRT. Additionally, a minority of women reported being ideally against the use of replacement hormones, were advised against using HRT by doctors, and did not use it because of the fear of side effects. We found a positive association between patient education, health care attitude, and HRT usage. A significant number of women knew about HRT from the media, and most of them were not informed by a health professional. Despite this, the interviewed doctors considered their knowledge about HRT as ‘good’ and would recommend HRT: only 5.6% would not prescribe it. *Conclusions*: Our results highlight the need for information about HRT among patients and health professionals, along with the need for more effective communication, evaluation, and suggestion of treatment.

## 1. Introduction

Menopause is the less explored season of women’s existence, in the literature and in everyday life. Scientifically, it results from the reduced secretion of estrogen and progesterone, which occurs as the finite store of ovarian follicles is depleted. Natural menopause is diagnosed after 12 months of amenorrhea [1,2]. The onset is not an abrupt change but is preceded by a transition state, the perimenopause, characterized by the gradual loss of oocytes, altered responsiveness to gonadal steroid feedback, wide hormonal fluctuations, and irregular menstrual patterns [3]. The menopause can be associated with many clinical manifestations: vasomotor symptoms; urogenital problems, such as vaginal dryness, itching, dyspareunia; and additional conditions, such as anxiety, mood changes, and sleep disturbances [4]. The prolonged lack of estrogen also affects the bones and the cardiovascular system and increases the risk of long-term consequences, such as osteoporosis [5].

Hormone Replacement Treatment (HRT) in menopause consists of the administration of estrogen, alone or associated to progesterone, aiming to relieve these uncomfortable fluctuating symptoms and to prevent the onset of pathologic conditions [6].

The current scientific evidence indicates that wisely selected HRT is generally useful and rarely dangerous [7]. Hormonal therapy is effective in the prevention of osteoporosis [8,9] and in the treatment of vulvovaginal atrophy [10]; it has also been shown to be the most effective treatment for vasomotor symptoms [11].

Nevertheless, the history of HRT has suffered many ups and downs. After a period of success at the end of the 20th century, when HRT seemed to be the answer to all women’s problems, things changed after the publication of the first large-scale study on its collateral effects: the Women’s Health Initiative (WHI) [12]. The WHI study, published in 2002, raised concerns about HRT and the health risks observed in post-menopausal users older than 60 years and/or women who have been post-menopausal for more than 10 years. In those patients, the use of HRT was associated with an increased risk of incident and fatal breast cancer [12,13]. The conclusion had an impressive mediatic resonance, although it was clearly based on some methodological inaccuracies. In fact, the enrolled women were asymptomatic, on average older than 60 years old, and frequently more than 10 years after the onset of menopause. These results struck the medical community, causing widespread distrust in HRT, with a dramatic drop in its prescription in the following years [14]. 

Subsequent follow-up of the studied cohorts and a comprehensive re-analysis of the data induced the elaboration of less catastrophic conclusions [15]. 

The history of HRT over the years has shown a trend with two peaks of utilization: a first rise in the 1960s and a second and higher increase in the years 1999–2000. After, there was a precipitous decline in HRT use in many countries [16], and this rate of utilization persists nowadays. The most prominent medical societies published numerous guidelines on the proper use and prescription of HRT, focusing mainly on adequate patient selection and route of administration. Apparently, this effort was not enough to settle the confusion among both health professionals and patients on the indications for HRT: a diffuse distrust and many uncertainties appear to persist among physicians [4,14,17].

The aim of this study is to examine the prevalence of HRT use in menopausal women living in a northern Italian city and women’s experience with menopause and HRT. This study also investigates the knowledge about HRT of general practitioners (GPs) and gynecologists of the same geographic area, and their attitude toward its prescription. 

## 2. Materials and Methods

This study was approved by the local Ethics Committee (protocol number AslVC.Med.18.01). We conducted a survey of 50–59-year-old women in Vercelli (Novara), an industrial city in the North of Italy with 170,000 inhabitants, located in Eastern Piedmont. At the conception of this study, the total number of women aged 50–59 years registered in this Local Health Unit was 13,767. 

**Figure 1:**
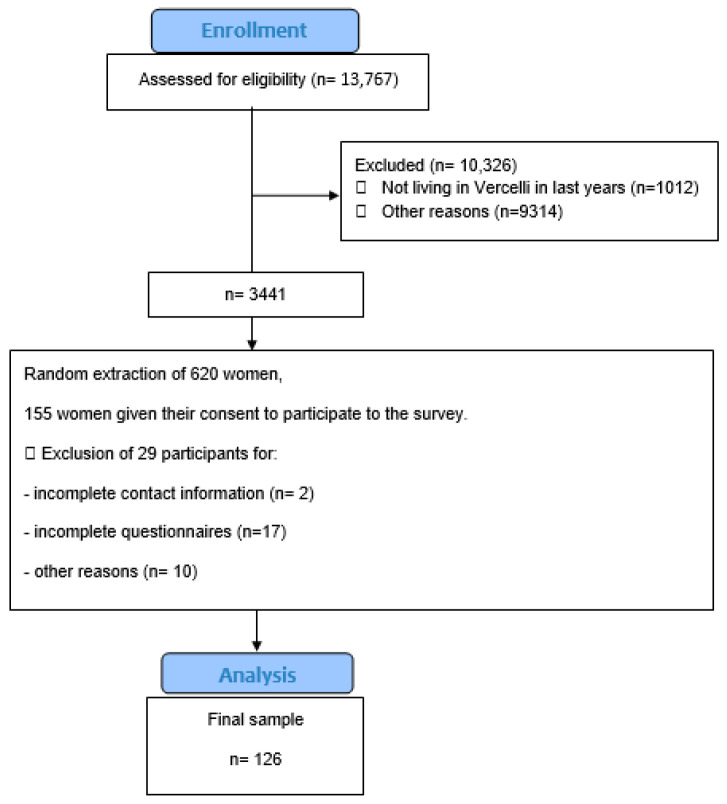
CONSORT flow chart.

We planned a cross-sectional population survey on a random sample of this population, on the basis of a previous study [16]. We conducted four rounds of phone calls in order to obtain 155 respondents willing to participate in the interview process. Out of these 155 individuals, 29 patients were excluded because failed to give correct contact information or to complete the questionnaire or for other reasons, resulting in a final sample size of 126 respondents (CONSORT flow chart, Figure 1). We also surveyed all GPs and gynecologists in the same area. 

We extracted the addresses and telephone numcbers of the participants and sent a presentation letter of the phone interview to them. We administered the questionnaire from May to July 2018, through a Computer-Assisted Telephone Interview. All phone calls were made by a single researcher. We introduced all the questionnaires with a standard sentence, describing this study and informing about data protection and about the possibility of dropping out from this study at any time. 

We designed the questionnaire on a model of those already validated and translated into Italian [16] (Appendix A). The questionnaire asked about menopausal status, women’s attitude towards HRT, use of HRT, sources of information on menopause and HRT, and socio-economic status. The questionnaire was organized into sections. One of the main outcomes of this study was HRT use throughout life and at the time of the interview. 

We also conducted a different survey by sending a questionnaire by email to all the 124 GPs and 29 gynecologists on the list of those practicing in the area (obtained from the local Medical Council). Questions were chosen based on L. Gao’s study (2017) [18] to investigate their attitude to prescribing HRT and their knowledge about it (Appendix A). Doctors were previously informed about this study by a letter. 

We tabulated the answers of women and doctors using Microsoft Excel 2011, in a pseudonymized way: a single researcher entered a code corresponding to each participant. We analyzed the sociodemographic, behavioral, and clinical characteristic distributions between HRT users and non-HRT users through Graph Pad 6. We tested binomial and categoric differences in study variables between the two groups using the Chi square test (or Fisher test as appropriate). We calculated differences in continuous variables between the two groups with a Student’s *t* test. 

The sample was obtained and evaluated by a single researcher. Since a response rate of 25% was expected, we extracted 620 subjects and contacted them until we finally reached 126 interviewed women (Figure 1). Our medical sample consisted of 54 doctors in total; 83.3% (n = 45) were GPs and 16.7% (n = 9) were gynecologists.

## 3. Results

In total, 101 interviewed women were in menopause. Twelve participants answered that were using or have used HRT in the past. The prevalence of HRT use in menopause was 11.9%, and the used formulations were 58.3% estrogens + progestinic, 33.3% Tibolone, and 16.7% estrogens + bazedoxifene (SERM), during a variable period of years. The average age at which the patients started assuming HRT was 49.1 years old (Table 1). Ninety-three women were aware of HRT, while thirty-three women were not informed about it (Table 1). 

In total, 41.7% of the users affirmed that they are satisfied with hormonal replacement therapy; 75.0% would recommend the treatment to a friend, while the remaining 25.0% would not, and 16.7% reported that they are unsatisfied with the treatment.

Table 1 shows the reasons why they did not choose HRT: most patients (38.3%) reported not having such intrusive symptoms and not needing any medical treatment, 23.5% of women reported being ideally against the use of replacement hormones, 8.6% of women were advised against using it by doctors, and 2.5% do not use HRT because afraid of the side effects.

Interestingly, of the 126 interviewed women, 73.8% reported knowing about the use of HRT: 92.1% of these patients were in menopause. A significant number of women who knew about HRT (31.2%) have received information about menopause from the media (TV, newspapers, radio, etc.), and most of them did not receive the information from a health professional: 50.5% have never received information from an expert (Table 2). Table 2 describes which categories are most likely to receive information about HRT. Women who had some health issues before menopause (*p* = 0.074), patients whit regular interaction with the GP (*p* = 0.014; 5.4 appointments/year vs. 3.1 appointments/year), and women familiar with breast cancer (*p* = 0.019) were more informed about HRT. 

Patients who usually have intake of vitamin D appeared to be more updated about HRT than the non-users (51.1% vs. 28.1%, *p* = 0.038). There is no difference in the two groups regarding the use of complementary medicines (Table 2).

Table 3 shows the results of the interview presented to the 54 doctors. The gender distribution was equal. The physicians generally considered their knowledge about HRT as ‘good’ (96.3%).

In total, 75.9% would recommend HRT: 18.5% (n = 10) would prefer to first discuss the case with a colleague, and only 5.6% (n = 3) would not recommend it. 

In most cases (75.9%), physicians prescribed oral treatment, and less frequently, transdermic (24.1%) or local assumption (27.8%). In total, 55.6% prescribed alternative options to HRT, such as Phytotherapy.

In total, 94.3% believed that HRT could be the most efficient treatment for the management of menopausal symptoms, and 100% believed that the general female population should receive more information on the positive aspects of hormonal therapy. Furthermore, 96.3% also answered that health workers should be more informed.

Regarding the WHI study, 67.9% affirmed that the study did not have any influence on clinical decisions, 28.6% that they prescribed HRT with more caution after the study results, and only one doctor (3.6%) did not recommend HRT due to the study. 

## 4. Discussion

Several longitudinal studies have investigated the prevalence of HRT use in menopause during the last decades. In the Nurses’ Health Study, it was reported that 15.8% of women used HRT during a 14-year observed period (1980–1994) [19]. In a Japanese study, the proportion of all the participants who had used HRT was 13.8% [20]. In a cohort study in Denmark, only 28.4% of the participants used HRT from 1995 to 2010 [21]. The proportion of women aged more than 54 years old who started HRT from 1990 to 2001 was 16.8%, and the proportion of women who started HRT at more than 55 years of age was 9.0% in a survey conducted in 2003 [22,23].

In Italy, only 4.0–7.0% of menopausal women use HRT: between these patients, most were gynecologists or gynecologists’ wives. It is a rare case in medicine in which doctors use a therapy more than patients [24].

In our survey, the proportion of women who used HRT is 11.9%, while only 3.0% were using it at the time of the survey, similarly to other Western industrialized countries.

In 2016, the International Menopause Society also recommended that the duration of HRT should not be limited, and that the long-term user rate has to be increased [25].

In total, 41.7% of the participants of our study affirmed to be satisfied with hormonal replacement therapy, and 75.0% would recommend the treatment to a friend, while the remaining 25.0% would not, and 16.7% reported that they were unsatisfied with the treatment. 

Of the 126 interviewed women, 73.8% affirmed that they know about the use of HRT: most of these patients were in menopause. In the literature, many studies confirm a variety of sources of information about menopause and HRT (e.g., healthcare providers, medical surveys, professional societies or hospitals, internet, TV, magazines, and friends and family) [26,27,28]. Unfortunately, a study published by Hamid et al. showed that most women had poor knowledge regarding menopause: 35.0% did not use treatments to relieve symptoms, and only 27.0% had good knowledge about HRT [27]. 

Chinese women, on the other hand, who were found to have good knowledge about menopause but poor knowledge about HRT, think that menopausal symptoms should not be treated [28]. Also, for most of our interviewed patients (38.3%), non-HRT users reported having symptoms that were not intrusive enough to justify the drug therapy.

The EMPOWER study, conducted on 1858 post-menopausal women, reported that concern about side effects is one of the main reasons for not using hormonal products [29]. In our results, only 2.5% do not use HRT because of the fear of side effects, and 23.5% of women affirmed being ideally against the use of replacement hormones.

Other works reported that most patients discontinued HRT after the WHI study publication because of the fear of side effects, even if they realize that HRT can alleviate disturbing symptoms [30]. In our research, doctors recommended not using HRT only to 8.6% of the interviewed women: our results could be considered encouraging data, assimilable to the great possibility of receiving information in an alternative, conscious, and independent way. 

In fact, we have found a positive association between patient education, health care attitude, and HRT usage. We observed higher assumption of HRT in case of more informed patients—about their health in general or for previous comorbidities- correlated to work occupation, and with better socio-economic status. 

Women appreciate being listened to and being informed about the possible approaches and about the risks and the effects of each treatment option on their quality of life. 

It is also important to stress that the effect of the education about HRT adherence was long-term rather than temporary [31].

This may be correlated with factors such as access to care, recognized risk for disease, health control, confidence levels, and other medical reasons [32]. It may also be possible that women with a higher socio-economic status have access to better health services. HRT was used 12.3 times more in university hospitals and 5.4 times more in private hospitals, when compared to public hospitals [32].

Consultation with doctors, GPs or gynecologists, is fundamental for the patient in the choice of HRT. In clinical practice, many specialists would theoretically favorably prescribe HRT, but very often they do not believe that this would be their responsibility [33]. 

In our study, 94.3% believe that HRT is the most efficient treatment for the management of menopausal symptoms, and 100.0% believe that the female population should receive more information on the positive aspects of hormonal therapy. In total, 96.3% of doctors also think that health workers should be more informed.

Moreover, in our sample, physicians generally considered their knowledge about HRT as ‘good’ (96.3%), and 75.9% would recommend HRT: only 18.5% would prefer to first discuss the case with a colleague, and only 5.6% would not recommend it. This is probably due to the evolution of studies and to the generational changes in the sample of health professionals, temporally less connected to the WHI study. In fact, 67.9% of them affirmed that this research did not have any influence on their clinical decisions.

GPs are officially in charge of patients’ integral wellness, and they are strategical actors in the promotion of a healthy lifestyle and preventive topics. In comparison to gynecologists, their comfort level in providing counseling regarding the risks, benefits, and alternatives to vaginal estrogen is not so strong, and this could be a knowledge gap [34]. 

Beyond this, as highlighted in our data, many factors seem to affect HRT usage. In fact, medical influence, place of residence, age, occupation, stipulation of a health insurance, concerns about the side effects, overall knowledge surrounding HRT, partner’s job, advice and influence of neighborhoods, and the hospital they applied for have been identified as factors affecting the rate of HRT in the performed analyses [32]. 

Our results could be useful to highlight the need for information about HRT among patients and health professionals, along with the need for more effective communication, evaluation, and suggestion of treatment.

Despite the interesting insights that our survey provides, we intend to underline some limitations, such as the sample size of HRT users, to be implemented in future studies: we expected an even higher patient response rate. Our research has the objective of being a pilot source of information describing women’s and doctors’ attitude to HRT. Our survey does not aim to define static conclusions on the subject but wants to trigger a possible dialogue on a very important topic for women’s health.

Another limitation is the lack of further analysis in the case of surgical menopause. Moreover, our attention was focused on an industrial populated Italian area, and the results could be different in poorer and less educated region of the world.

## 5. Conclusions

Our data support the need for successful communication and shared decision making, allowing patients and medical professionals to work together and to consider the best scientific evidence for health care decisions.

Our study does not aim to define rigid conclusions but wants to trigger a possibility of discussion on a very important topic for women’s health: this survey is a preliminary descriptive analysis of patients’ and doctors’ attitude to HRT.

In particular for menopausal patients, doctors have the task of sharing medical evidence, including information about the benefits and risks of HRT. They should also be conscious of potential prejudices and make efforts to minimize the impact of them.

## Figures and Tables

**Table 1:** Menopause and HRT in our population sample (main results).

Answer Options		Results	Percentages
Women in menopause		101	80.2%
Age * (mean value, in years) of the sample		55.0	
Age at menopause * (mean value, in years)		50.0	
Type of menopause	Physiological	91/101	90.1%
	Surgically induced	6/101	5.9%
	Pharmacologically induced	3/101	3.0%
	After radiotherapy	1/101	1.0%
Experience of menopause related symptoms	Yes	69/101	68.3%
	No	32/101	31.7%
Did you have information about HRT?	Yes	93/101	92.1%
	No	8/101	7.9%
Are still using or have used HRT in the past?	Yes	12/101	11.9%
	No	89/101	88.1%
Have used HRT for more than 6 months?	Yes	10/101	9.9%
	No	91/101	90.1%
Currently using HRT	Yes	6/101	5.9%
	No	95/101	94.1%
If you know about HRT, why don’t you use/have used HRT?	Heath issues/contraindications Preferred not to interfere with the natural life cycle Did not have any discomfort Doctors advised against it Afraid of side effects Waiting for prescription	18/81 ° 19/81 31/81 7/81 2/81 4/81	22.2% 23.5% 38.3% 8.6% 2.5% 4.9%
***If you use/have used HRT (12 patients)***			
Onset age of use (mean value, in years)		49.1	
Type of HRT	Tibolone	4/12	33.3%
	Estrogens + bazedoxifene	2/12	16.7%
	Estrogens + progestinic	7/12	58.3%
Way of administration	Oral	10/12	83.3%
	Transdermic	3/12	25.0%
	Topical	-	-
Prescribed by	Gynaecologist	11/12	91.7%
	GP	1/12	8.3%
Prescribed for	1 month	1/12	8.3%
	6 months	1/12	8.3%
	1 year	1/12	8.3%
	5 years	7/12	58.3%
	>10 years (until 55 years)	1/12	8.3%
	Not remember	1/12	8.3%
Therapy duration	Less than a year	6/12	50.0%
	1 to 2 years	3/12	25.0%
	3 to 4 years	1/12	8.3%
	4 to 5 years	1/12	8.3%
	More than 5 years	1/12	8.3%
Described effect *	Positive	12/12	100.0%
	Negative	3/12	25.0%
	Other	8/12	66.7%
Perception of adequate information	Yes	8/12	66.7%
	No/ I don’t know	4/12	33.3%
Intention to stop HRT	Yes	4/12	33.3%
	No/ don’t know	8/12	66.7%
Have stopped taking HRT	Yes	6/12	50.0%
	No	6/12	50.0%
≥1 clinical check-up/year	Yes	9/12	75.0%
	No/ doesn’t remember	3/12	25.0%
Level of satisfaction	Not satisfied	2/12	16.7%
	Quite satisfied	5/12	41.7%
	Very satisfied	5/12	41.7%
Would recommend HRT to a friend	Yes	9/12	75.0%
	No/ doesn’t know	3/12	25.0%
Side effects	Yes	7/12	58.3%
	No	5/12	41.7%
Complications	Yes	2/12	16.7%
	No	10/12	83.3%

GP = General Practitioner. ° 81 women know about HRT and not use the therapy. * More than one possible answer.

**Table 2:** Variables of HRT users: informed versus not-informed women (main results).

Variables		Informed about HRT (n: 93 Participants)		Not Informed about HRT (n: 33 Participants)		*p*-Value
Age (mean value, in years)		55.0		54.3		0.195
Socio- economic status (SES)	Low SES	27/93	29.0%	17/33	51.5%	0.083
	Medium SES	53/93	56.9%	13/33	39.4%	
	High SES	13/93	24.5%	3/33	9.1%	
Occupation	Housewife	15/93	16.1%	7/33	21.2%	0.324
	Freelance or employee	64/93	68.8%	24/33	72.7%	
	Manager or professional	8/93	8.6%	-	-	
	Unemployed	5/93	5.4%	1/33	3.0%	
	Retired	1/93	1.1%	1/33	3.0%	
Place of birth	North Italy	68/93	73.1%	22/33	66.7%	0.226
	Central Italy	4/93	4.3%	-	-	
	South Italy	13/93	13.9%	4/33	12.1%	
	Abroad	8/93	8.6%	7/33	21.2%	
Currently in menopause	Yes	77/93	82.8%	24/33	72.7%	0.216
	No	16/93	17.2%	9/33	27.3%	
Type of menopause	Spontaneous	68/77 °	73.1%	23/24 #	95.8%	>0.999
	Surgically induced	5/77	6.5%	1/24	4.2%	
	Pharmacologically induced	3/77	3.9%	-	-	
	Caused by radiotherapy	1/77	1.3%	-	-	
Onset of menopause (mean value, in years)		49.9		50.1		0.857
Menopausal symptoms	Yes	59/93	63.4%	21/33	63.6%	0.984
	No	34/93	36.6%	12/33	36.4%	
Do you receive information regarding menopause?	Yes	71/93	76.3%	22/33	66.7%	0.277
	No	22/93	23.7%	11/33	33.3%	
Who gave information about menopause *?	GP	4/93	4.3%	-	-	0.572
	Gynaecologist	14/93	15.1%	4/33	12.1%	0.78
	Pharmacist/informative meetings	2/93	2.2%	-	-	>0.999
	TV/radio/papers	25/93	26.9%	3/33	9.1%	0.049
	Internet	33/93	35.5%	8/33	24.2%	0.284
	Friends/relatives/colleagues	33/93	35.5%	13/33	39.4%	0.689
	Others (homeopath, education, endocrinologist)	4/93	4.3%	-	-	
The information was given	As an active offer	23/93	24.7%	5/33	15.1%	0.059
	After it was requested	24/93	25.8%	4/33	12.1%	
	No information was given	46/93	49.4%	24/33	72.7%	
Currently relevant health issues	Yes	45/93	48.4%	14/33	42.5%	0.65
	No	48/93	51.6%	18/33	54.5%	
	No answer	-	-	1/33	3.0%	
Health issues before menopause	Yes	46/93	49.5%	10/33	30.3%	0.074
	No	47/93	50.5%	22/33	66.6%	
	No answer	-	-	1/33	3.0%	
Familiarity for breast cancer	Yes	28/93	30.1%	3/33	9.1%	0.019
	No/ don’t remember	65/93	69.9%	29/33	87.9%%	
	No answer	-	-	1/33	3.0%	
Attended recommended screenings	Yes	85/93	91.4%	31/33	94.0%	0.445
	No	8/93	8.6%	1/33	3.0%	
	No answer	-	-	1/33	3.0%	
Physical activity	Less than 2 h a week	33/93	35.5%	13/33	39.4%	0.837
	Between 2 and 5 h a week	21/93	22.5%	5/33	15.1%	
	More than 5 h a week	37/93	39.8%	11/33	33.3%	
	Do not practice	2/93	2.2%	4/33	12.1%	
Number of GP’s appointments /year		5.43		3.1		0.014
Perception of one’s health	Bad	1/93	1.1%	-	-	0.481
	Quite good	58/93	62.3%	17/33	51.5%	
	Excellent	33/93	35.5%	15/33	45.4%	
	No answer	1/93	1.1%	1/33	3.0%	
Vitamin D assumption	Yes	47/93	50.5%	9/33	27.3%	0.038
	No	45/93	48.4%	23/33	69.7%	
	No answer	1/93	1.1%	1/33	3.0%	
Use of complementary medicines	Yes	24/93	25.8%	8/33	24.2%	>0.999
	No	68/93	73.1%	23/33	69.7%	
	No answer	1/93	1.1%	1/33	3.0%	

GP = General Practitioner. ° 77 patients in menopause. # 24 patients currently in menopause. * More than one possible answer.

**Table 3:** Doctors’ answers to the questionnaire regarding HRT (main results).

Questions		Results	Percentages
Medical Specialization	Gynecology	9/54	16.7%
	GP	45/54	83.3%
Sex	Female	29/54	53.7%
	Male	25/54	46.3%
Competence in prescribing HRT	Low	1/54	1.9%
	Sufficient	52/54	96.3%
	Excellent	1/54	1.9%
Took part in courses regarding HRT	Yes	15/54	27.8%
	No	39/54	72.2%
Would happily attend a course	Yes	46/54	85.2%
	No	8/54	14.8%
Is HRT necessary for women with menopausal symptoms?	I agree	3/54	5.6%
	I agree in most cases	40/54	74.1%
	It is necessary only in a few cases	11/54	20.4%
	No, totally disagree	-	-
Would you recommend HRT to a patient who has never taken it before (with no contraindications)?	Yes, I would	41/54	75.9%
	I would first seek the advice of some colleague	10/54	18.5%
	No, I wouldn’t	3/54	5.6%
Which way of administration do you usually recommend? *	Oral	41/54	75.9%
	Transdermic	13/54	24.1%
	Topical	15/54	27.8%
Do you believe that patients should be more informed regarding risks and benefits of HRT?	Yes	54/54	100.0%
	No	-	-
Do you believe that health workers should be more informed regarding risks and benefits of HRT?	Yes	52/54	96.3%
	No	2/54	3.7%
Reasons why there is a scarce use of HRT in Italy *	Patients are often not aware of HRT	17/54	31.5%
	GPs are often not aware of usefulness of HRT	15/54	27.8%
	Gynecologists are often not aware of usefulness of HRT	6/54	11.2%
	Prejudices about HRT	10/54	18.5%
	Contrasting opinions regarding HRT	34/54	63.0%
	Not confident when prescribing HRT	21/54	38.9%
	Risks and side effects of HRT	9/54	16.7%
Can patients become dependent on HRT?	Yes	11/54	20.4%
	No	42/54	77.8%
	No answer	1/54	1.8%
Are there possible alternatives?	Yes	30/54	55.6%
	No	24/54	44.4%
If yes, what alternatives do you offer?	Herbal medicines	17/30 °	56.7%
	Supplements to improve the lipidic balance	10/30	33.3%
	Homeopathic medicines	1/30	3.3%
	Naturopathy	2/30	6.7%
You believe that these alternative therapies…	Can be complementary to HRT	22/30	40.7%
	Have a minor risk/better benefit ratio	6/30	11.1%
	Are not comparable to benefits of HRT	23/30	42.6%
	No answer	3/30	5.5%
Do you know about the WHI trial?	Yes	17/54	31.5%
	No/ don’t remember	37/54	68.5%
Has your approach/ tendency to prescribe HRT been influenced by the WHI trial?	No, I was not influenced	19/54	35.1%
	Yes, now I prescribe HRT with much more caution	8/54	14.8%
	Yes, I don’t prescribe HRT any more	1/54	1.8%
	No answer	26/54	48.1%
**In your opinion, HRT:**			
Is the most effective treatment for menopausal symptoms?	Yes	51/54	94.4%
	No	3/54	5.6%
Has a good protective effect on the cardiovascular system?	Yes	30/54	55.5%
	No answer	3/54	5.5%
	No	21/54	38.8%
Is a good treatment to prevent bone density loss?	Yes	45/54	83.3%
	No answer	2/54	3.7%
	No	7/54	13.0%
Has a good impact on urogenital atrophy?	Yes	53/54	98.1%
	No	1/54	1.9%
Has a good preventive impact on some cancers?	Yes	22/54	40.7%
	No answer	3/54	5.6%
	No	29/54	53.7%

GP = General Practitioner. ° 30 doctors answered yes. * More than one possible answer.

## Data Availability

Data are available on request from corresponding author.

## Appendix A. Questionnaire

### Appendix A.1. Questionnaire for Women

Use of hormone replacement therapy in menopause

-1st SECTION: PERSONAL DATA

1. Age: ______ years old
2. Marital status:
  - Nubile
  - Married/cohabiting
  - Widow
  - Separate
3. Degree:
  - No school
  - Elementary school
  - Lower secondary school
  - High school
  - Degree
  - 3rd level qualification (doctorate or master)
4. Profession:
  - Freelance work (e.g., private lawyer/ doctor/ architect)
  - Self-employment (e.g., trader, craftsman)
  - Employee work/managerial roles (e.g., doctor, lawyer, teacher, professor university)
  - Employee work/executive roles (e.g., office worker, sales assistant, secretary)
  - Housewife (occupied only at home)
  - Retired
  - Unemployed
  - Other…. (specify)
5. Country of origin: ________________________________________________
6. Province of birth: _____________________________________________
7. Province of residence: ___________________________________________

-2nd SECTION: MENOPAUSAL STATE OF WOMEN

8. Are you currently in menopause? (if not, skip to question 13)
  - Yes
  - No
9. Age of onset of menopause? _______years
10. Is it a menopause:
  - Natural
  - Surgical
  - By irradiation
  - Pharmacological
11. Do you suffer or have you suffered from menopause-related disorders?
  - Yes
  - No
  - I don’t know/I don’t remember
12. Define for each of the following symptoms a score from 1 to 5 depending on how much the symptom in question occurred in your specific case (1 corresponds to ‘‘not yes presented’, 5 corresponds to ‘‘a lot’):
  - Flushes of heat sweating…..
  - Sleep disorders….
  - Menstrual problems….
  - Irritability/ changes of humor/ difficulty of concentration or to remember things….
  - Tiredness….
  - Depression….
  - Increase of weight….
  - Articular and pelvic pains….
  - Urinary disorders….
  - Irritation and vaginal dryness….
  - Reduction of sexual desire…
  - Other (describe what and express your perception with a score 1-5)……
13. Have you ever heard of HRT? Have you ever received any information about this? (if not, skip to question 16)
  - Yes
  - No
  - I don’t remember
14. Are you currently taking or have you previously taken HRT?
  - Yes
  - No
15. If you know, but don’t take HRT: Why did you opt not to assume it?....

-3rd SECTION: WOMEN’S EXPERIENCE—INFORMATION ON MENOPAUSE

16. According to you, menopause is:
  - A normal transitional phase of a woman’s life, of limited duration
  - A syndrome requiring pharmacological treatment
17. In your opinion, does menopause cause disturbances? (read all, only one possible)
  - Yes, to all women
  - It depends on the woman
  - No, it does not cause discomfort
18. Have you ever received information about menopause? (if no, skip to question 22)
  - Yes
  - No
  - I don’t know/ I don’t remember
19. On what aspects did you receive information? (read all, all possible)
  - Menopause in general
  - Symptoms of menopause
  - Drugs to be used in menopause
  - Natural-type products for use in menopause
  - Other ….(specify)
20. From whom did you receive information about menopause? (read all, all possible)
  - General practitioner
  - Gynecologist
  - Pharmacist
  - Courses/meetings at the Hospital/Family planning clinic
  - Homeopath / natural medicine centers
  - Newspapers
  - Television and/or radio
  - Internet
  - Voluntary/consumer associations
  - Friends/relatives/acquaintances/colleagues
  - Other… (specify)
21. In general, information received about menopause from a healthcare professional (doctor, pharmacist, etc.):
  - Given directly without your asking
  - Need to ask them
  - You have not received information from a healthcare professional
22. According to your knowledge, what are the disturbances caused by menopause? (all answers are possible)
  - Hot flashes
  - Sweating
  - Sleep disturbances
  - Menstrual problems
  - Irritability/mood changes
  - Difficulty to concentrate/difficulty to remember things
  - Tiredness
  - Depression
  - Weight gain
  - Joint and pelvic pain
  - Urinary disturbances
  - Vaginal irritation and dryness
  - Decreased sexual desire
  - No disturbance
  - Other …. (specify)
23. In general, what aspects of menopause would you like to know more about? (all possible)
  - What it is
  - Health Risks
  - Possible therapies
  - Other…. (specify)

If you have never heard of HRT skip to 6th SECTION

-4th SECTION WOMEN’S EXPERIENCE—INFORMATION ABOUT HRT.

(You answered you had information about HRT.)

24. What aspects have you been informed about? (all possible)
  - Possible Risks
  - Benefits and treatment of menopause symptoms
  - Prevention of some diseases related to the menopause period
  - Menopause checkups and tests (Blood pressure, blood tests, bone densitometry, etc.)
  - Other…. (specify)
25. Who told you about it first? (all possible)
  - General practitioner
  - Gynecologist
  - Media (radio, television, internet, newspapers)
  - Friends and relatives
  - Scientific journals
  - Pharmacist
  - Working environment
  - Other…. (specify)
26. Overall, please describe how satisfied you are with the information on HRT you received under a scale from 1 (‘‘I’m not satisfied’’) to 5 (I’m very satisfied) ………….
27. Have you received conflicting information about HRT?
  - Yes
  - No
  - I don’t remember
28. On what aspects have you received conflicting information? (all possible)
  - Possible risks
  - Benefits and treatment of menopause symptoms
  - Prevention of some diseases related to the menopause period
  - Other…. (specify)
29. In general, what aspects of HRT would you like to know more about? (all possible)
  - Benefits
  - Risks
  - Recruitment methods
  - Other…. (specify)
30. What motivated you to inquire about HRT?
  - Awareness of entering or having entered menopause
  - Previous hysterectomy with or without bilateral oophorectomy
  - Appearance of menopausal symptoms with repercussions on quality of life
  - On medical advice

If not taking HRT skip to 6th SECTION: ANAMNESIS AND HEALTH STATUS.

If you take/have taken HRT:

-5th SECTION: WOMEN’S EXPERIENCE: HRT USE

31. At what age did you start taking HRT? _______ years
32. What type of HRT have you taken?
  - Estrogen only
  - Estrogen + progestinic
  - Tibolone
  - Other ….
33. What route of administration?
  - Oral route
  - Transdermal route (patch)
  - Transcutaneous route (topical, gel or cream)
34. Trade name of drug: ________________________
35. How long were you told to assume it? _months; ____ years;
36. How long have you been taking HRT?
  - Less than 1 year
  - Between 1 and 2 years
  - Between 2 and 3 years
  - Between 3 and 4 years
  - Between 4 and 5 years
  - More than 5 years
37. Who prescribed HRT for you?
  - General practitioner
  - Gynecologist
  - Other specialist.... (specify)
38. What information did the prescriber give you? (all possible)
  - The benefits
  - The disadvantages
  - The risks
  - Other ….(specify)
39. Do you remember some of them? ….(to specify)
40. Do you think you have been adequately informed?
  - Yes
  - No
  - I don’t know
41. On which indication have you been prescribed HRT? (all are possible)
  - Reduce vasomotor symptoms related to menopause
  - Prevent osteoporosis and bone fractures
  - Reduce the risk of colorectal diseases
  - Improve the lipoprotein values
  - Improve glucose metabolism
  - Reduce cardiovascular risk related to post-menopause
  - Prevent and treat urogenital atrophy
  - Prevent connective tissue atrophy
  - Treat muscle/joint pain
  - Treat mood changes
  - Treat sleep/wake disorders
  - Treat changes in the sexual sphere
  - Other (specify)
42. Have you ever thought about stopping HRT treatment?
  - Yes
  - No
43. Have you ever stopped taking HRT?
  - Yes
  - No
44. If yes, why did you interrupt the treatment?
  - Spontaneously, in relation to the resolution of symptoms
  - On the advice of a doctor or other healthcare professional
  - For fear of side effects
  - By occurrence of side effects
  - Because I have had little benefit
  - Other ….(specify)
45. Do you or have you performed a clinical check-up at least once a year during assumption?
  - Yes
  - No
  - I don’t remember
46. Please rate your satisfaction with HRT treatment, relatively menopausal symptoms on a scale of 1 (‘‘I’m not at all satisfied’’) to 5 (‘‘I am very satisfied’’)……………
47. Based on your experience, give your opinion on the following statements based on a scale from 1 (‘‘I do not agree’’) to 5 (‘‘I fully agree’’). The HRT:
  - It is a good solution if a woman has several disturbances …………….
  - It is a good solution even if a woman has few disturbances………………
  - It must be avoided……………………
  - It is a good solution to prevent health disorders age-related…………………….
  - It has many side effects…………………
48. Would you advise a friend of yours who suffers from menopause to use HRT?
  - Yes
  - No
  - I don’t know
49. Have you developed side effects from HRT? (all possible)
  - Breast swelling or pain
  - Reappearance of heavy menstrual-like flows
  - Abnormal bleeding
  - Headache
  - Weight gain
  - Nausea
  - Water retention
  - Irritability
  - Depression
50. Have you developed complications potentially related to HRT? (all possible)
  - Venous thromboembolism
  - Gallstones
  - Endometrial cancer
  - Breast cancer
  - Cardiovascular pathologies
  - Cerebrovascular disorders

-6th SECTION: ANAMNESIS AND HEALTH STATUS

51. Do you currently have any pathologies?
  - Yes, which ones? _____________________________________________________________________
  - No
  - I don’t know/I don’t remember
52. Have you ever had any health problems before menopause?
  - Yes, which ones? _____________________________________________________________________
  - No, no problem
  - I don’t know/I don’t remember
53. Have you family history of breast cancer?
  - Yes
  - No
  - I don’t know/I don’t remember
54. Have you family history of ovarian cancer?
  - Yes
  - No
  - I don’t know/I don’t remember
55. Do you periodically perform age-recommended screenings?
  - Yes
  - No
  - I don’t know/I don’t remember
56. Which ones? (all answers are possible, read all)
  - Mammography every two years (every year if on HRT)
  - Screening for colorectal cancer
  - Pap test
  - Checking the lipid/cholesterol profile
  - Blood sugar
  - Transvaginal ultrasound
  - Bone densitometry
57. What was your weight at the following ages (excluding pregnancies):
  - Around 30 years old: ___________ kg
  - Around 50 years old: ___________ kg
58. Current height: ________ cm
59. Smoking Habit:
  - Never smoker
  - Smoker
  - Former smoker (stopped more than a year ago)
60. If you are a smoker or a former smoker:
  - Less than 10 cigarettes/day
  - 10–19 cigarettes/day
  - More than 20 cigarettes/day
61. How would you describe your level of physical activity regarding your job?
  - Very tiring
  - Tiresome
  - Standing
  - Sedentary
62. Still talking about physical activity, in your spare time you practice:
  - Less than 150 min/week of moderate physical activity or less than 75 min/week of vigorous physical activity or combinations of the two
  - Between 150 and 300 min/week of moderate physical activity or between 90 min and 150 min per week of vigorous physical activity or combinations of the two
  - More than 300 min/week of moderate aerobic physical activity or more than 150 min of intense physical activity
63. How many times a year do you contact the general practitioner on average?.................................
64. In general, compare your health status to women of the same age, on a scale of 1 (‘‘poor’’) to 5 (‘‘excellent’’): ……………
65. What have you used to avoid the symptoms of menopause and osteoporosis?
  - Adequate diet
  - Physical activity
  - Adequate dietary and/or supplemental Calcium intake
  - Vitamin D
  - Selective estrogen receptor modulator (SERM)
  - Bisphosphonates
  - Denosumab
  - Strontium Ranelate
  - Nothing
66. Do you use complementary medicines to treat menopause? (if not, end)
  - Phytotherapy (red clover, Cimicifuga Racemosa, soy and soy derivatives, etc.)
  - Acupuncture
  - Traditional Chinese Medicine (moxibustion, Tuina massage, dietetics, Chinese pharmacopoeia, etc.)
  - Naturopathy
  - Homeopathy
  - Manipulative techniques
  - Yoga
  - Relaxation techniques
67. Who recommended complementary medicine to you?
  - Gynecologist/GP
  - I knew it because I had treated other previous disturbs
  - Friends or colleagues
  - I asked, because medical treatment was not enough

--------------------------------------------------------------------------------------------------------------

### Appendix A.2. Questionnaire for Gynecologists and General Practitioners

-1st SECTION: PERSONAL DATA

1. Indicate the type of specialization:
  - Gynecology
  - General Practitioner
2. How many years have you been in the profession?.......
3. Year of birth……
4. Gender
  - Female
  - Male
  - Other

-2nd SECTION: ATTITUDE AND KNOWLEDGE ABOUT RISKS E BENEFITS OF HRT

5. How do you rate your personal knowledge of HRT on a scale of 1 (corresponds to ‘‘poor’’) to 5 (‘‘excellent’’)?.......................
6. Would you be willing to take further training on HRT?
  - Absolutely, I also consider it necessary
  - Yes
  - I don’t think it’s strictly necessary, since they haven’t been done great improvements in this area
  - No
7. ‘‘Hormone replacement therapy is necessary for women who complain of climacteric symptoms’’. How do you approach this statement?
  - I strongly agree
  - I agree, in most cases
  - It is only needed in a few cases
  - I absolutely disagree
8. Would you recommend HRT to a woman suitable for treatment for the first time?
  - Yes, I would recommend it
  - I should first compare myself with other colleagues
  - No, I would not recommend it
9. In daily clinical practice, to how many post-menopausal women do you recommend the HRT?
  - 0%
  - 10%
  - 20%
  - 30%
  - 40%
  - 50%
  - 60%
  - 70%
  - 80%
  - 90%
  - 100%
10. In daily clinical practice, in percentage terms, to how many post-menopausal women do you prescribe HRT?
  - 0%
  - 10%
  - 20%
  - 30%
  - 40%
  - 50%
  - 60%
  - 70%
  - 80%
  - 90%
  - 100%
11. What route of administration do you usually prescribe?
  - Oral route
  - Transdermal route
  - Transcutaneous route
12. Do you consider necessary to train patients more about risks and benefits of HRT?
  - Yes, by standardizing and clarifying the information and denying not so reliable studies
  - Yes, it would be useful that the patient has already clear ideas about HRT
  - No, the information provided on an outpatient basis is sufficient to opt for a suitable choice
13. Could be necessary to further train healthcare personnel about risks and benefits of HRT?
  - Yes
  - No
14. In your opinion, why does Italy have a low prevalence of HRT prescription? (More than one answer is possible)
  - Lack of patients’ awareness
  - Lack of Gynecologists’ awareness
  - Lack of General Practitioners’ awareness
  - Traditional views
  - Side Effects
  - Dissemination of Conflicting Information
  - Scarce doctors’ propensity to prescribe it
15. Regarding the benefits of HRT, what is your opinion respect to the following statements? True or false
  - I believe it is the most effective treatment for menopausal symptoms ……
  - I think it has a good cardioprotective effect ……
  - I think it is an effective treatment to prevent bone loss………..
  - I think it has a good positive impact on urogenital atrophy…..
  - I think it has a good preventive impact against some tumors……

-Based on your knowledge, select one option for each of the following, regarding the RISK deriving from the use of HRT:

16. Vasomotor symptoms
  - Very common
  - Common
  - Rare
  - Unmodified
17. Breast cancer
  - Very common
  - Common
  - Rare
  - Unmodified
18. Endometrial cancer (in relation to continuous combined estrogen-progestinic therapy)
  - Very common
  - Common
  - Rare
  - Unmodified
19. Ovarian cancer
  - Very common
  - Common
  - Rare
  - Unmodified
20. Lung cancer
  - Very common
  - Common
  - Rare
  - Unmodified
21. Colorectal cancer
  - Very common
  - Common
  - Rare
  - Unmodified
22. Venous thromboembolic events
  - Very common
  - Common
  - Rare
  - Unmodified
23. Abnormal bleeding
  - Very common
  - Common
  - Rare
  - Unmodified
24. Risk of pathological fractures
  - Very common
  - Common
  - Rare
  - Unmodified
25. Cardiovascular disturbs
  - Very common
  - Common
  - Rare
  - Unmodified
26. Diabetes Mellitus
  - Very common
  - Common
  - Rare
  - Unmodified
27. Ischemic stroke
  - Very common
  - Common
  - Rare
  - Unmodified
28. Hemorrhagic stroke
  - Very common
  - Common
  - Rare
  - Unmodified
29. Alzheimer’s disease
  - Very common
  - Common
  - Rare
  - Unmodified
30. Dementia
  - Very common
  - Common
  - Rare
  - Unmodified
31. Depression
  - Very common
  - Common
  - Rare
  - Unmodified
32. Weight gain
  - Very common
  - Common
  - Rare
  - Unmodified
33. Can patients develop addiction to the therapy?
  - Yes
  - No
  - I don’t know/I don’t remember
34. Do you usually offer other alternatives to HRT for the treatment of menopausal symptoms?
  - Yes, which ones? (to specify)…………………
  - No
35. If yes, you believe that these methods:
  - Have a better risk/benefit ratio than HRT
  - Are in no way comparable to the benefits offered by the HRT
  - May be a complementary aid to the HRT

-3rd SECTION: IN 2002, FOLLOWING AN AMERICAN PUBLICATION° THERE HAS BEEN AN IMPORTANT DEBATE IN ITALY RESPECT THIS KIND OF THERAPY

36. Have you ever heard of the WHI study?
  - Yes
  - No
  - I don’t know/I don’t remember
37. If yes, do you remember why the study was interrupted? (more answers are possible)
  - Increased cardiovascular risk
  - Increased thromboembolism events
  - Increased vasomotor symptoms
  - Increase in breast cancer
  - Increase in endometrial cancer
  - Increased ovarian cancer
  - Increased colorectal cancer
  - Increase in lung cancer
  - Increase in abnormal uterine bleeding
  - Increased risk of pathological bone fractures
  - Increase in urinary incontinence symptoms
  - Increased prevalence of diabetes mellitus
  - Increase in ischemic stroke
  - Increase in hemorrhagic stroke
  - Increase in Alzheimer’s disease
  - Increase in dementia
  - Increased depression
  - Weight gain
  - Drug addiction
38. If yes, do you remember which aspects the discussion focused on?
  - Effects of therapy on the cardiovascular system
  - Cancer risk
  - Risk of bone fractures
  - Other…. (to specify)…………….
39. Your approach and tendency to prescribe HRT was influenced by the results of the WHI study?
  - Yes, I do not prescribe HRT
  - Yes, I now prescribe HRT but with much more caution
  - No, I was not influenced by this study
40. Are you currently updated on new studies that have partially refuted the findings of the WHI study? Can relate some of the reasons why the study was retained inappropriate? (Multiple answers are possible)
  - The study included a very large number of subjects with characteristics and comorbidities that interfered with the study objectives
  - Many of the patients in the study have assumed HRT for first time more than 10 years after the onset of menopause
  - The study assimilated (and did not specify) the identified risks concerning the only estrogen therapy compared to those related to estrogen-progestinic
  - Other.…(specify)………………………………

° Writing Group for the Women’s Health Initiative Investigators. Risks and Benefits of Estrogen Plus Progestin in Healthy Postmenopausal Women: Principal Results From the Women’s Health Initiative Randomized Controlled Trial [12].
